# Improved antimalarial activity of caprol-based nanostructured lipid carriers encapsulating artemether-lumefantrine for oral administration

**DOI:** 10.4314/ahs.v20i4.20

**Published:** 2020-12

**Authors:** Paul Achile Akpa, Joseph Abuchi Ugwuoke, Anthony Amaechi Attama, Chinenye Nnenna Ugwu, Ezinwanne Nneoma Ezeibe, Mumuni Audu Momoh, Adaeze Chidiebere Echezona, Franklin Chimaobi Kenechukwu

**Affiliations:** 1 Department of Pharmaceutics, University of Nigeria, Nsukka; 2 Department of Pharmaceutical Microbiology and Biotechnology, University of Nigeria, Nsukka

**Keywords:** Nanostructured lipid carriers, artemether-lumefantrine, malaria, Caprol

## Abstract

**Background:**

Artemether and lumefantrine display low aqueous solubility leading to poor release profile; hence the need for the use of lipid-based systems to improve their oral bioavailability so as to improve their therapeutic efficacy.

**Aim and objective:**

The objective of this work was to utilize potentials of nanostructured lipid carriers (NLCs) for improvement of the oral bioavailability of artemether and lumefantrine combination and to evaluate its efficacy in the treatment of malaria. This study reports a method of formulation, characterization and evaluation of the therapeutic efficacies of caprol-based NLC delivery systems with artemether and lumefantrine.

**Method:**

The artemether-lumefantrine co-loaded NLCs were prepared using the lipid matrix (5% w/w) (containing beeswax and Phospholipon® 90H and Caprol-PGE 860), artemether (0.1%w/w) and lumefantrine (0.6%w/w), sorbitol (4%w/w), Tween® 80(2%w/w as surfactant) and distilled water (q.s to 100%) by high shear homogenization and evaluated for physicochemical performance. The in vivo antimalarial activities of the NLC were tested in chloroquine-sensitive strains of Plasmodium berghei (NK-65) using Peter´s 4-day suppressive protocol in mice and compared with controls. Histopathological studies were also carried out on major organs implicated in malaria.

**Results:**

The NLC showed fairly polydispersed nano-sized formulation (z-average:188.6 nm; polydispersity index, PDI=0.462) with no major interaction occurring between the components while the in vivo study showed a gradual but sustained drug release from the NLC compared with that seen with chloroquine sulphate and Coartem®. Results of histopathological investigations also revealed more organ damage with the untreated groups than groups treated with the formulations.

**Conclusion:**

This study has shown the potential of caprol-based NLCs for significant improvement in oral bioavailability and hence antimalarial activity of poorly soluble artemether and lumefantrine. Importantly, this would improve patient compliance due to decrease in dosing frequency as a sustained release formulation.

## Introduction

Malaria, a common parasitic vector-borne poverty-related disease caused by the bite of infected mosquitoes, affects the quality of life of millions of people in malaria endemic regions of the world and is the leading cause of mortality and morbidity in the developing world 1. Many obstacles lie in the face of the effective control of malaria, including the plethora of antigenic variations coupled with antigenic polymorphism 2 and wide range of mechanisms by means of which the causative agent evades the host immune system, which hinders vaccine production efforts^3^. Furthermore, efforts at vector control have only yielded marginal results.

The resistance of Plasmodium falciparum to most of the widely employed antimalarial agents such as sulphadoxine-pyrimethamine, chloroquine, mefloquine and amodiaquine sets further limits to disease elimination efforts. The oral bioavailability of most antimalarials is low 4. The rising incidence of parasite resistance and the paucity of novel drug candidates in the pipeline of the global pharmaceutical sector makes it necessary for the exploitation of other modes of drug formulation with the objective of developing drug delivery systems with desirable features that can culminate in safe and effective medicines with better therapeutic outcomes utilizing the currently available active pharmaceutical ingredients (APIs). Nanomedicines could help to overcome the limitations of currently utilized antimalarial agents, including low intracellular concentrations^5^ and toxicity 6.

The World Health Organization (WHO) strongly recommends the use of artemisinin-based combination therapies (ACTs) in a bid to overcome the development of resistance to the drugs by the malaria parasite 7. Artemether-lumenfantrine (ARM-LFN) combination is one of the most popular combinations and the first line fixed-dose ACTs prescribed by the WHO for oral treatment of uncomplicated malaria 8. In this combination, artemether exerts rapid onset of action though of short duration of action whereas lumefantrine exhibits a relatively slower action and it has a longer halflife and thus persists longer in the systemic blood circulation^9^. Artemether effects an almost instantaneous symptomatic relief as it brings about a reduction in the parasite burden while lumefantrine neutralizes any parasite remnants 10. The use of these two agents together forecloses contact of the parasites with either of the drugs alone thus reducing the likelihood of resistance developing to these agents. Combination products operate synergistically to effect a reduction in gametocyte carriage and this further hinders transmission of the causative parasite. It has been established that the artemether-lumefantrine combination has poor aqueous solubility as well as low oral bioavailability 11.

Nanocarriers have been shown to enhance dissolution of poorly soluble drugs owing to their large surface area^12^. Lipid based drug delivery systems (LBDDS) boost oral bioavailability of lipophilic drugs via numerous mechanisms 13–16. First, the ingestion of LBDDS triggers contractions of the gall bladder and elicits an increase in biliary and pancreatic secretions like bile salts, cholesterol and phospholipids. These secretions together with gastric shear lead to the formation of a crude emulsion which maintains the drug in a solubilized form affording better gastrointestinal absorption^17^. The surfactants and co-surfactants present in the LBDDS further augment the solubility of poorly soluble drugs^18^. Secondly, it is established that some excipients including surfactants such as Tween^®^ 80 and Solutol^®^HS 15 when present in a LBDDS restrain intestinal cytochrome P450 enzymes which catalyze pre-systemic metabolism 19. Certain lipids containing large chain fatty acids or those with a high degree of unsaturation bring on lymphatic uptake of LBDDS which facilitates avoidance of the hepatic first-pass metabolism of the drug in question 20.

NLCs are second generation of LBDDS, and they were chosen due to their advantages such as enhanced drug loading, cost effectiveness, nontoxicity over the other types of lipid carriers such as liposomes, nanoemulsions, microemulsions and solid lipid nanocarriers 17. The purpose of an NLC formulation is to produce particles in which the oil is incorporated into the core of the solid lipid and the drug is solubilized in the oily core. This should result in a higher loading capacity, encapsulation efficiency, and controlled drug release as the drug dissolves in the oil and simultaneously encapsulates in the solid lipid, which should also lead to slower polymorphic transition and lower crystallinity index (higher stability) 18. Furthermore, co-loaded nanoparticles are preferred over the single drug-loaded nanoparticles because co-loading has been shown to increase the therapeutic efficiency of drugs by increasing protection rate, reducing the drug dosage, etc. 17. Although we have developed LBDDS of artemether and lumefantrine in our earlier studies 21–23, to the best of our knowledge, there is paucity of information in the literature on the use of phospholipid-modified caprol-based NLC for co-delivery of artemether and lumefantrine. Consequently, the purpose of this work was to formulate and evaluate a nanostructured lipid carrier (NLC) using a combination of solid lipids (beeswax and Phospholipon^®^ 90 G) and a liquid lipid (Caprol PGE 860) for the entrapment of the lipophilic drugs artemether and lumefantrine. The NLCs produced were characterized to assess fundamental physicochemical attributes such as the particle size and zeta potential, morphology, thermal properties, compatibility and stability. The in vivo antimalarial study was performed in Wistar mice infected with Plasmodium berghi using Peter´s protocol while haematological properties as well as the histopathology of key organs of the mice were evaluated.

## Materials and methods

### Materials

Artemether was obtained from Hangzhou Dayang Chemical Co. Ltd. (Zhejiang, China), while lumefantrine was sourced from Guilin Pharmaceutical Co Ltd. (Shanghai, China). Phospholipon^®^ 90H (P90H) (Phospholipid GmbH, Köln,, Germany), sorbitol (Caesar &Loretz, Hilden, Germany), sorbic acid (Foodchem Int. Co., China), beeswax (Carl Roth, Karlsruhe, Germany), Polysorbate 80 (Tween^®^ 80) (Acros Organics, Geel, Belgium), Capryol-PGE 860 (Abitech Corp, USA), Coartem^®^ (Novartis, Basel, Switzerland) and distilled water (Lion water, University of Nigeria, Nsukka, Nigeria) and other solvents and reagents were used as procured from their manufacturers without further purification.

Adult albino Wistar mice of both sexes infected with Plasmodium berghei(NK 65) malaria parasites were employed in the study.

### Methods

#### Preparation of lipid matrix for nanostructured lipid carrier formulation

Lipid matrix was prepared by the fusion method 21 using beeswax and Phospholipon^®^ 90H (as solid lipids) in combination with caprol-PGE 860 (as liquid lipid). The solid lipids and liquid lipid were used at 7:3 ratio (i.e. 21.0 g of BW/P90H admixture and 9.0 g of caprol-PGE 860). First of all, the solid lipids (at 7:3 ratio of BW and P90H) (21.0 g of beeswax and 9.0 g of P90H) were weighed, added in a glass beaker placed inside an oil bath (liquid paraffin) and melted together in the temperature-regulated bath at a temperature of 70 °C. The mixture was stirred continuously until a homogenous, transparent colourless melt was obtained. The homogenous mixture of the lipid matrix was stirred further at room temperature until solidification. After 24 hours, this lipid matrix was melted in the temperature-regulated bath at a temperature of 80 °C followed by addition of 9.0 g (8.98 ml) of caprol-PGE 860. The mixture was stirred continuously until a homogenous, transparent white melt was obtained. The homogenous mixture of the lipid matrix was stirred at room temperature until solidification. The lipid matrix was then stored in airtight and moisture resistant glass bottle in the refrigerator until used.

#### Preparation of drug-loaded lipid matrix

Drug-loaded lipid matrix was prepared by fusion^22^ using the lipid matrix, artemether and lumefantrine. With target lipid concentration of 5.0 %w/w and target drug concentrations of 0.1 %w/w of artemether and 0.6 %w/w of lumefantrine in the nanostructured lipid carrier (NLC) to be developed, 5 g of the lipid matrix (LM3) was melted in the temperature-regulated oil bath at a temperature of 80 °C followed by addition of 0.1 g of artemether and 0.6 g of lumefantrine. The mixture was stirred continuously until a homogenous, transparent white melt was obtained. The homogenous mixture of the drug-loaded lipid matrix was stirred further at room temperature until solidification. It was then stored in airtight and moisture resistant glass bottle in the refrigerator until used.

#### Differential scanning calorimetry (DSC) analysis of plain and drug-loaded lipid matrices

Melting transitions and changes in heat capacity of the lipid matrix of beeswax and P90H structured with Caprol-PGE 860, artemether, lumefantrine and drug-loaded lipid matrix were determined using a differential scanning calorimeter (DSC Q100 TA Instrument, Germany).

A 5 mg quantity of each sample was weighed into an aluminum pan, hermetically sealed and the thermal behaviour determined in the range of 20 to 350 °C at a heating rate of 5 °C/min. The temperature was held at 80 °C for 10 min and thereafter, cooled at the rate of 5 to 10 °C/min. Baselines were determined using an empty pan, and all the thermograms were baseline-corrected.

#### Fourier transform infra-red (FT-IR) spectroscopic analysis of plain and drug-loaded lipid matrices

FT-IR spectroscopic analysis was conducted on the lipid matrix of beeswax and P90H, Caprol-PGE 860, lipid matrix structured with Caprol-PGE 860, artemether, lumefantrine and drug-loaded lipid matrix of beeswax and P90H structured with Caprol-PGE 860 using a Shimadzu FT-IR 8300 Spectrophotometer (Shimadzu, Tokyo, Japan) and the spectrum was recorded in the wavelength region of 4000 to 400 cm-1 with threshold of 1.303, sensitivity of 50 and resolution of 2 cm-1 range. A smart attenuated total reflection (SATR) accessory was used for data collection. The potassium bromate (KBr) plate used for the study was cleaned with a tri-solvent (acetone-toluene-methanol at 3:1:1 ratio) mixture for baseline scanning. A 0.1 g quantity of each sample was mixed with 0.1 ml nujol diluent. The solution was introduced into the potassium bromate (KBr) plate and compressed into discs by applying a pressure of 5 tons for 5 min in a hydraulic press. The pellet was placed in the light path and the spectrum obtained. Spectra were collected in 60 s using Gram A1 spectroscopy software, and the chemometrics were performed using TQ Analyzer^1^.

#### Preparation of nanostructured lipid carrier (NLC)

Caprol-based nanostructured lipid carrier (NLC) encapsulating artemether and lumefantrine were prepared using the drugs, lipid matrix, Polysorbate^®^ 80 (Tween^®^ 80) (mobile surfactant), sorbitol (cryoprotectant) and distilled water (vehicle) by the high shear hot homogenization method 21–23 (5%w/w of the LC (lipid matrix) formulation) was placed in glass beaker and melted at 80 °C on the temperature-regulated hotplate (IKA instruments, Germany) and the drugs (0.1 %w/w of artemether and 0.6 %w/w of lumefantrine) were added to the melted lipid matrix. Aqueous surfactant solution consisting of sorbitol (4 %w/w) and Polysorbate^®^ 80 (2 %w/w) was prepared in a beaker and heated at the same temperature. The hot aqueous surfactant phase was then dispersed in the hot lipid phase (oily phase) using a high-speed homogenizer (Ultra-Turrax T25) (IKA-Werke, Staufen, Germany) at 1000 rpm for 5 min. The obtained pre-emulsion was homogenized at 15,000 rpm for 30 min, and allowed to cool or re-crystallize at room temperature. The formulation composition of the NLC is shown in Table 1.

**Table 1:** Optimized formula for the preparation of the nanostructured lipid carrier

S/No	Ingredient	% w/w
1	Lipid matrix (Beeswax + Phospholipon 90H+Caprol-PGE 860)	5.0
2	Artemether/lumefantrine	0.1/0.6
3	Polysorbate^®^ 80 (Tween^®^ 80)	2.0
4	Sorbitol	4.0
5	Distilled water	q.s. to 100

#### Characterization of the nanostructured lipid carrier (NLC)

##### Particle size and polydispersity index

Mean diameter, Z. Ave (nm) and polydispersity index (PDI) of the NLC were measured using a zetasizer nano-ZS (Malvern Instrument, Worceshtire, UK) equipped with a 10mw He-NE laser employing the wavelength of 633 nm and a backscattering angle of 173° at 25 °C. The sample was diluted with double-distilled water to obtain a suitable scattering intensity, before photon correlation spectroscopic (PCS) analysis. Zeta potential was also determined.

##### Compatibility study by Fourier transform infra-red (FT-IR) spectroscopy for the NLC formulation

Fourier transform infra-red (FT-IR) spectroscopic analysis using a Shimadzu FT-IR 8300 Spectrophotometer (Shimadzu, Tokyo, Japan) and the spectrum was recorded in the wavelength region of 4000 to 400 cm-1 with threshold of 1.303, sensitivity of 50 and resolution of 2 cm-1 range. A smart attenuated total reflection (SATR) accessory was used for data collection. The potassium bromate (KBr) plate used for the study was cleaned with a tri-solvent (acetone-toluene-methanol at 3:1:1 ratio) mixture for baseline scanning. A 0.1 ml volume of the NLC was mixed with 0.1 ml nujul diluent. The solution was introduced into the potassium bromate (KBr) plate and compressed into discs by applying a pressure of 5 tons for 5 min in a hydraulic press. The pellet was placed in the light path and the spectrum obtained. Spectra were collected in 60 s using Gram A1 spectroscopy software, and the chemometrics was performed using TQ Analyzer^1^.

##### Antimalarial and hematological studies on the formulations

Firstly, 20 adult albino Wistar mice of both sexes were procured, housed, and fed normally to acclimatize to the laboratory environment of the Faculty of Veterinary Medicine, University of Nigeria, Nsukka. The parasite, a chloroquine-sensitive strain of Plasmodium berghei NK 65 which was maintained in mice, was obtained from the Nigerian Institute of Medical Research (NIMR), Yaba, Lagos. Evaluation of the curative potential (in vivo anti-plasmodial activity) of the formulations against established plasmodium infection was carried out according to standard protocols established by Peter et al. 24. Briefly, the mice were divided into four groups of five mice each. Blood of the donor mice was collected by cardiac puncture and diluted with physiological saline (normal saline) to give a concentration of 1 x 10^8^ parasitized erythrocytes per mL. Ab initio, on day 0 of the test, percentage parasitemia of the donor mice was determine by Giemsa-stained thin blood smear of the donor mice. A 0.2 mL volume of the donor mouse erythrocyte equivalent to 2×10^7^ parasitized erythrocytes was injected intraperitoneally into each of the experimental mice on day 1. All the mice were inoculated with chloroquine sensitive strain of Plasmodium berghei (NK 65) and left untreated until the third day post inoculation to ensure establishment of plasmodium infection.

Post inoculation, all treatments were given orally per day for 3 days (day 4 to day 6). On day 1 of treatment (day 4), mice from the negative control group were administered with 0.2 mL/kg body weight of distilled water, while mice from the first positive control group received 10 mg/kg body weight of chloroquine phosphate tablet dispersed in distilled water, then 5 mg/kg on day 2 and 3 of treatment (day 5 and day 6). Mice from the second positive control group received 4 and 24 mg/kg of commercial fixed combination dose of artemether-lumefantrine (1:6) (coartem^®^) once daily. The other group received NLC formulation (M0) containing 4 and 24 mg/kg of artemether and lumefantrine (1:6). These dosages have been employed by previous researchers who evaluated the antimalarial activity of solid lipid nanoparticles loaded with artemether and lumefantrine in *Plasmodium berghei*-infected mice (21). The animals were randomized equally into four groups as follows:

Group 1: infected control (negative control) (infected but untreated)

Group 2: infected and treated with chloroquine phosphate (first positive control)

Group 3: infected and treated with coartem^®^ (second positive control)

Group 4: infected and treated with NLC (M0).

Parasitemia was determined 3, 7 and 14 days after treatment. Each mouse was tail-bled and a thin blood film was made on a microscope slide. The films were stained with 10 % Giemsa solution and examined microscopically to monitor the parasitemia level. Percentage parasitemia count in each animal was calculated using equation 1. Thereafter, mean parasitemia in each group was determined. The antimalarial activity in each group was determined using equation 2.

Eqn 1 $$\mathrm{Parasitemia}\;\mathrm{count}\;\mathrm{in}\;\mathrm{each}\;\mathrm{animal}\text{ (\%) = }\frac{\text{N}o\;\mathrm{of}\;\mathrm{infected}\;\mathrm{RBC}}{\mathrm{Total}\;\mathrm{no}\;\mathrm{of}\;\mathrm{RBC}}\times 100$$

Eqn 2 $$\mathrm{Reduction}\;\mathrm{in}\;\mathrm{parasitemia}\text{ (\%) = }\frac{\mathrm{Mean}\;\mathrm{pretreatment}\;\mathrm{parasitemia}\text{ - }\mathrm{Mean}\;\mathrm{posttreatment}\;\mathrm{parasitemia}}{\mathrm{Mean}\;\mathrm{pretreatment}\;\mathrm{parasitemia}}\times 100$$

The weight of the animals, hemoglobin concentration (Hb) and packed cell volume (PCV) were also monitored before inoculation, after inoculation, before treatment and after treatment up to the 14^th^ day after treatment. Moreover, the survivability of mice treated with the formulations was also determined.

##### Histopathological (histological) studies on the formulations

The mice were sacrificed on the fifteenth day post treatment and the liver and kidney of a mouse from each group subjected to histological studies. Tissue sections of the liver and kidney of mouse from each group were fixed in 10 % normal saline and dehydrated in ascending grades of ethanol. Thereafter, the tissues were cleared in chloroform overnight, infiltrated and embedded in molten paraffin wax. The blocks were later trimmed and sectioned at 5–6 mm. The sections were deparaffinized in xylene, rinsed with water and subsequently stained with Haematoxylin and Eosin (H and E) and fixed for viewing which was done with a moticam (D-MOTICAM 580, U.S) fitted to the polarized photomicroscope.

##### Storage stability studies of the formulations

The NLC formulation was also subjected to time-resolved pH analysis for three months to check the effect of storage on the stability of the NLC formulation. The pH of the formulation was determined using a pH meter (Suntex TS-2, Taiwan) after one day, one week, one month and three months of storage at room temperature (28 ± 3 °C).

## Statistical analysis

All experiments were performed in replicates for validity of statistical analysis. Results were expressed as mean ± SD. ANOVA and Student's t-test were performed on the data sets generated using SPSS. Differences were considered significant for p-values <0.05.

## Results and discussion

**Figure 1:**
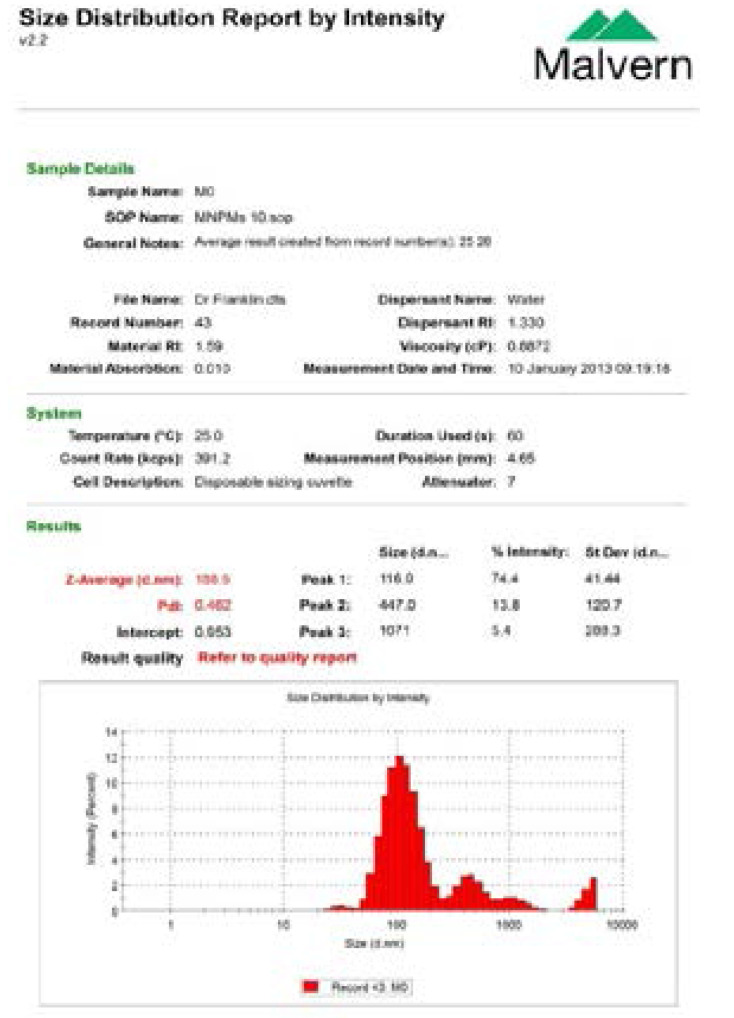
Photon correlation spectroscopy (PCS) analysis result of caprol-based nanostructured lipid carrier loaded with artemether and lumefantrine.

Nanostructured lipid carriers (NLCs) are an attractive approach for the delivery of highly lipophilic drugs such as artemether and lumefantrine as NLCs have advantages over other colloidal systems – solid lipid microparticles, solid lipid nanoparticles, liposomes, nanoemulsions, and microemulsions 25. This is because the majority of drugs have higher solubility in liquid lipids (oils) rather than solid lipids. The purpose of NLC formulation is to produce particles in which the oil is incorporated into the core of the solid lipid and the drug is solubilized in the oily core. This should result in a higher loading capacity, encapsulation efficiency, and controlled drug release as the drug dissolves in the oil and simultaneously encapsulates in the solid lipid; which should also lead to slower polymorphic transition and lower crystallinity index (higher stability) 25. Beeswax is a hard fat and a high melting point lipid for modified oral dosage forms, in addition to being a consistency agent (thickener) for topical formulations. Phospholipon^®^ 90H (P90H) is a phospholipid and an amphiphilic surface modifier used for oral, parenteral and topical formulations. Combination of lipophilic and hydrolipophilic (amphiphilic) surfactants yields better stabilization of dispersed systems. Consequently, beeswax was structured with a phospholipid, P90H, whereas caprol served as the liquid lipid to improve the solubilization of the drugs – artemether and lumefantrine. High oil content of NLC has been associated with less crystallinity 26. Caprol-based nanostructured lipid carrier (NLC) encapsulating artemether and lumefantrine were prepared using the drugs, lipid matrix, Polysorbate^®^ 80 (Tween^®^ 80) (mobile surfactant), sorbitol (cryoprotectant) and distilled water (vehicle) by the high shear hot homogenization method. NLC produced had average particle size of 188±6 nm and polydispersity index (PDI) of 0.462 ±4. Such a small particle size was obtained by precluding destabilization owing to creaming and controlling sedimentation caused by Brownian motion^27^. Details of the particle size analysis are shown in Figure 1. The PDI value of 0.462 is substantially high. It shows that the NLC is fairly polydispersed and indicates a broad particle size distribution 28. This is in agreement with the findings of other workers 29–35.

**Figure 2:**
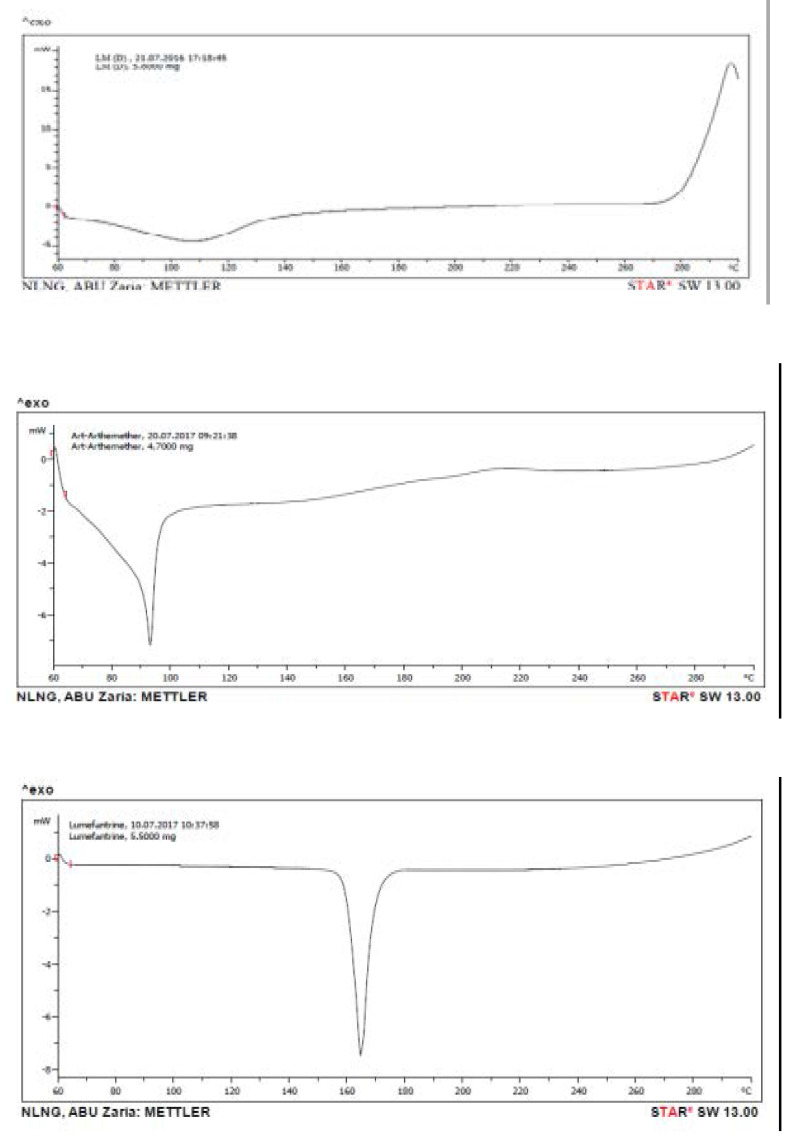

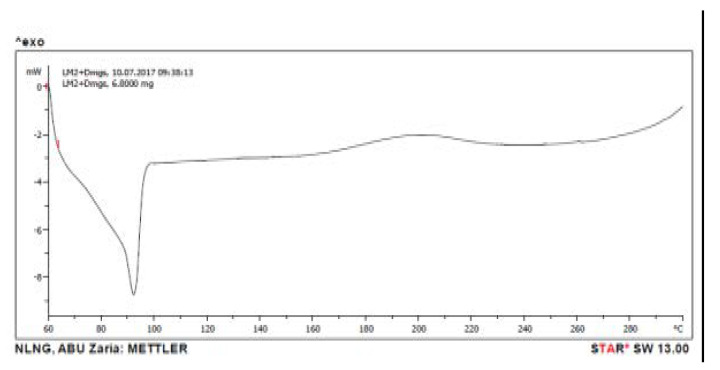
Differential scanning calorimetry (DSC) thermogram of (a) structured Phospholipid-modified beeswax-based lipid matrix structured with caprol-PGE 860 (LM_3_), (b) artemether, (c) lumefantrine, and (d) artemether and lumefantrine co-loaded lipid matrix.

The results of thermal analysis are as shown in Table 2 and Figures 2a-d. From Table 2, it could be seen that the individual drugs and lipid components had higher melting peak, artemether (92.5 °C), lumefantrine (163.4°C), but that of drug-loaded lipid matrix reduced to (91.8 °C). The lower enthalpy of drug loaded lipid matrix suggests that the formulation can produce matrix of lower crystallinity. Reduction in enthalpy generally suggests less crystallinity of lipid matrices 36–41. In effect, this will culminate in greater bioavailability of the drug since it will exhibit better dissolution properties.

**Table 2:** Thermal properties (DSC profiles) of drugs, plain and drug-loaded lipid matrices

Sample	Melting peak (°C)	Enthalpy (mW/mg)	Type of peak
	107.5	-4.5	Endothermic
LM_3_	278.3	2.1	Exothermic
Artemether	92.5	-7.2	Endothermic
Lumefantrine	163.4	-7.5	Endothermic
LM3+Drugs	91.8	-8.9	Endothermic

### Fourier transform infra-red (FT-IR) spectroscopic analysis of plain and drug-loaded lipid matrices

**Figure 3:**
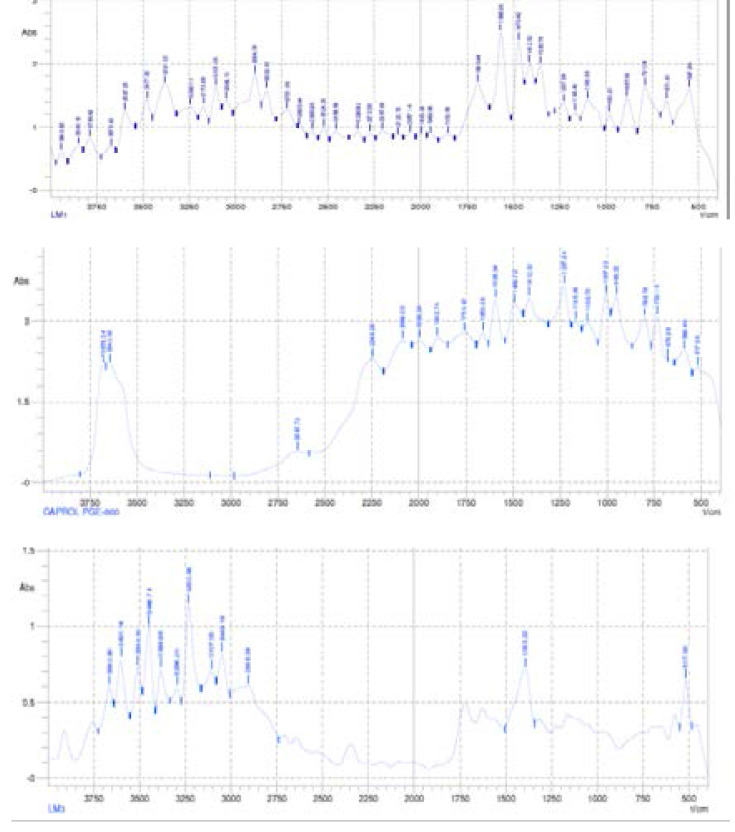
Fourier transform infra-red (FT-IR) spectrum of (a) lipid matrix based on beeswax and Phospholipon^®^ 90H (P90H) (LM_1_), (b) caprol-PGE 860 and (c) Phospholipid-modified beeswax-based lipid matrix structured with caprol-PGE 860 (LM_3_).

**Figure 4:**
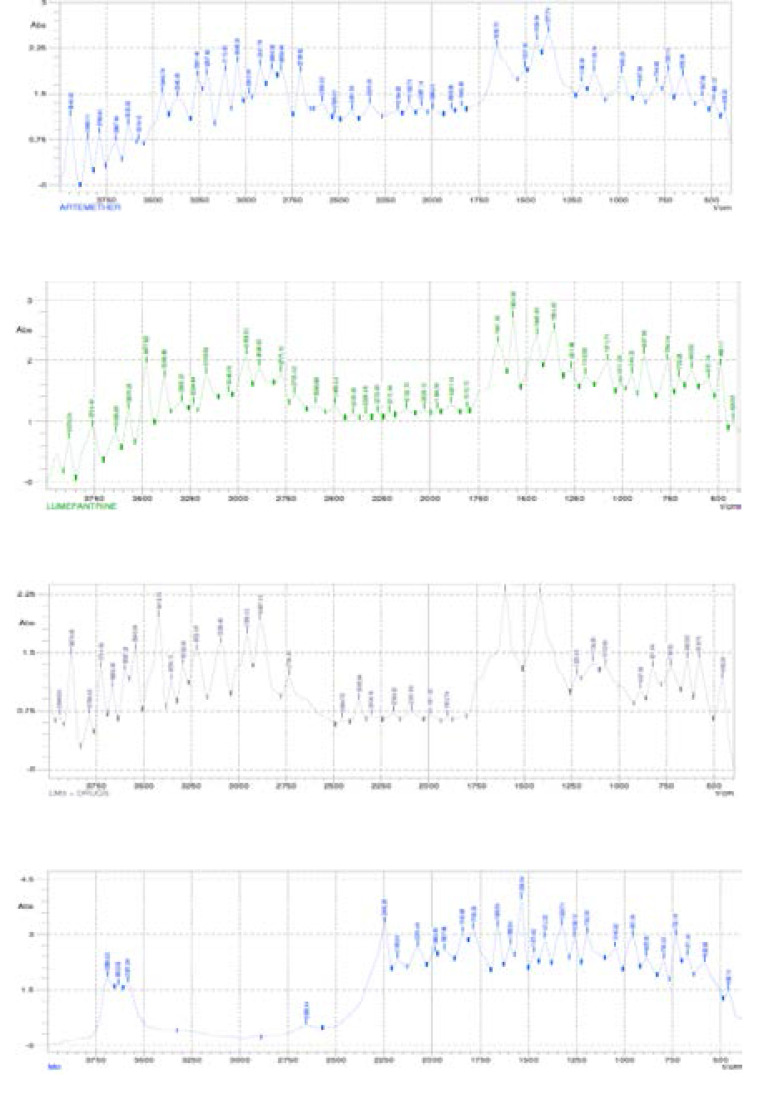
Fourier transform infra-red (FT-IR) spectrum of (a) artemether, (b) lumefantrine, (c) lipid matrix loaded with artemether and lumefantrine and (d) caprol-based NLC formulation containing artemether and lumefantrine.

Table 3 shows the compilation of the FT-IR results presented in Figures 3 and 4. It shows the major functional groups of the lipid constituents, the individual drugs, drug-loaded lipid matrix and that of the formulation. With all the major peaks which were seen in each of these components still showing even after the drug incorporation is an indication that the drug and the excipients are compatible since no major interaction occurred between the functional groups of the various components hence the appearance of the major peaks in the FT-IR spectrum. The overall FT-IR spectra of the drugs and all the excipient used in the formulation suggested absence of any incompatibility between the drug and the excipients 31.

**Table 3:** FT-IR profiles of excipients, drugs and formulations

Material	Principal peak (cm^-1^)	Type of bond
LM1	3381.12 3107.06 2894.76 1690.44 1566.92 1470.42 887.56 547.88	O-H bond vibration N-H bond vibration Carboxylic acid C-OH vibration\ C=C bond vibration Conjugated C=C bond vibration -CH3 bond bending Aromatic C-H out of plane bend C-Br bond vibration
Caprol-PGE 860	3678.34, 3643.60 1593.94 1227.24 1007.22 798.78, 733.16	O-H stretching Aromatic C=C bond vibration -C=C- stretching Amine group C-N bond vibration C-Cl bond vibration
LM_3_	3601.14, 3446.74 3230.58, 3049.16 1393.22 517.00	O-H bond vibration N-H bond vibration C-O bond vibration C-Br bond vibration
Artemether	3940.82 3110.92 3045.30 2705.62 1655.70 1377.78, 1130.74	O-H stretching N-H stretching -C-H streching O=C-H stretching C=O vibration C-O vibration
Lumefantrine	3759.40 3477.62 2956.52 1566.92 1443.40 1354.62 1076.70 887.56 486.12	O-H stretching N-H stretching O=C-H stretching Aromatic C=C bond vibration -CH3- bond bending C-O vibration Amine group C-N bond vibration Aromatic C-H out of plane bend C-Cl bond vibration
LM_3_+Drugs	3879.06, 3724.66 3419.72, 3222.86 2956.52 2736.50 1597.80 1412.52 821.94 578.76 455.24	O-H stretching N-H stretching -C-H stretching O=C-H stretching Aromatic C=C bond vibration -CH3- bond bending Aromatic C-H out of plane bend C-Br bond vibration C-Cl bond vibration
NLC (M_0_)	3689.92 2246.28 1783.08 1659.56 1536.04 1412.52 1323.74 1045.82 887.56	O-H stretching C=N stretching Vinyl ester bond vibration C=C bond vibration Aromatic C=C bond vibration -CH3- bond bending C-O vibration Amine group C-N bond vibration Aromatic C-H out of plane bend

### Antimalarial activity of the NLC

This shows the effectiveness of the treatments in eradicating parasitemia. From the results obtained as shown in Table 4, it was observed that the formulation that showed the same result as the standard treatment was more effective than chloroquine and the untreated control. On day 3 post inoculation, a mouse died in the groups treated with NLC (Mo), coartem^®^ and chloroquine phosphate but after the initiation of therapy, NLC(Mo) and coartem^®^ were able to prevent further loss of the animals but two more and four animals died in the groups treated with chloroquine phosphate and distilled water, respectively. This is consistent with a recent study where *Plasmodium berghei*-infected mice treated with artemether and lumefantrine co-loaded NLCs showed better antimalarial activity with respect to survivability period 17.

**Table 4:** Survivability in mice treated with caprol-based nanostructured lipid carrier

Batch/Group	Number of mice that survived			
	Day 3 after inoculation	Day 3 after treatment	Day 7 after treatment	Day 14 after treatment
NLC (M_0_)	4/5	4/5	4/5	4/5
Coartem^®^	4/5	4/5	4/5	4/5
Chloroquine phosphate	4/5	4/5	4/5	2/5
Distilled water	5/5	5/5	4/5	1/5

**Figure 5:**
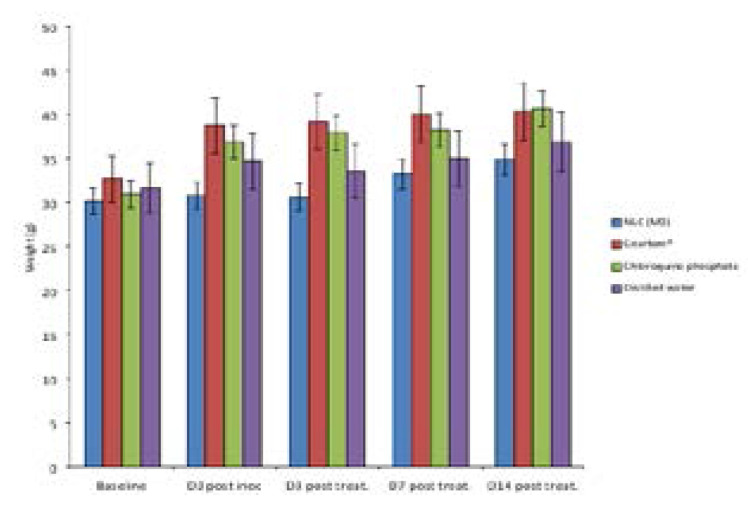
Effect of the formulations on the weight of the mice.

Figure 5 indicates the ability of the treatment to handle the worsening state of malaria in the mice which manifests as an increase in the weight (due to increase in the size of spleen, liver and possibly other blood-forming tissues). From the result, coartem^®^ appeared to be more effective in this task followed by Mo. Even though weight of animals in this group all increased, it was at a lower rate when compared to the groups treated with chloroquine and distilled water.

### Hematological properties of the NLC

**Figure 6:**
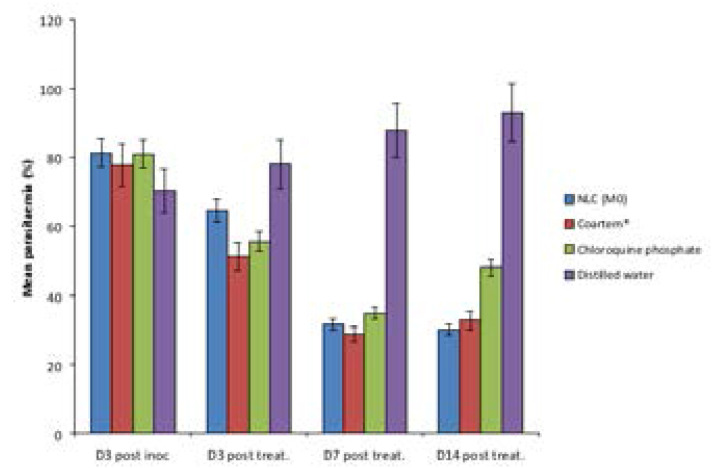
Mean percentage parasitemia in each group of the mice.

**Figure 7:**
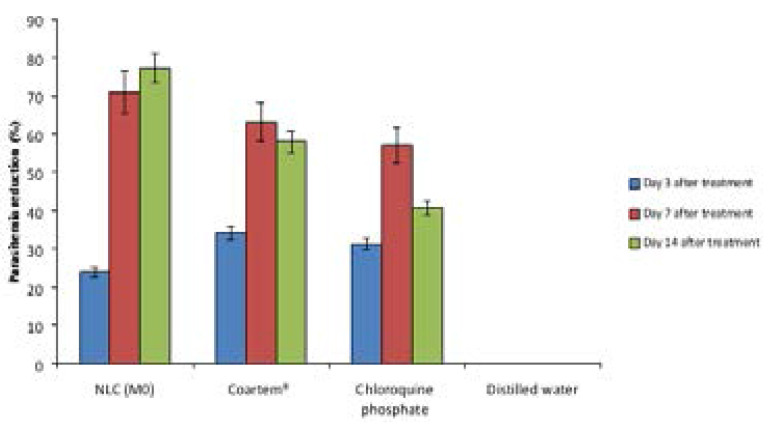
Percentage parasitemia reduction in the treatment groups.

**Figure 8:**
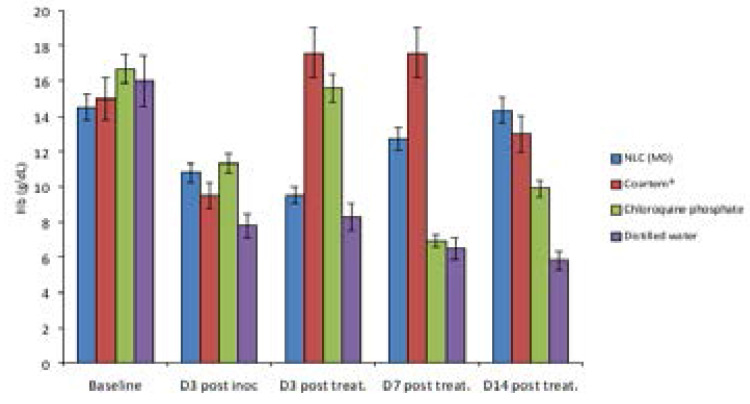
Effect of the formulations on hemoglobin of the mice.

**Figure 9:**
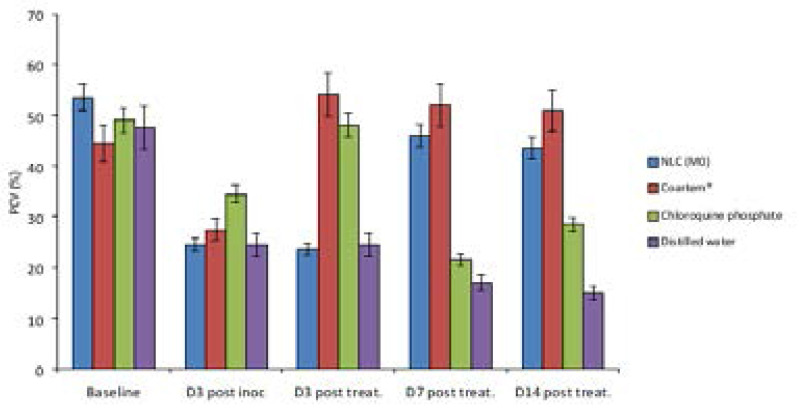
Effect of the formulations on packed cell volume of the mice.

From the result of the overall hematological studies carried out on the animals (Figures 6–9), it was noticed that the formulations (coartem^®^, chloroquine and Mo) were able to continually bring down the parasitemia level unlike what was observed in the untreated group. Also, from the result, it was observed that coartem^®^ and chloroquine appeared to be more drastic at the early stage of treatment but the NLC (Mo) proved to be much more efficacious. The coartem^®^ and chloroquine were more drastic in the reduction of parasitemia, restoration of normal hemoglobin level (12.0–17.5g/dl) after 14 days post-treatment and restoration of normal packed cell volume but at a point after days post treatment, recrudescence set in and both the hemoglobin and packed cell volume started to decrease after day 7 post-treatment and the parasitemia level in the group treated with coartem^®^ and chloroquine started to increase, while the level in the group treated with Mo group continued to decrease. This may be as a result of NLC formulation being able to sustain the release of the drugs in the body much more than the other two drug formulations, which is an advantage over the standard, as has been demonstrated in previous studies, where *Plasmodium berghei*-infected mice treated with artemether and lumefantrine co-loaded NLCs showed better antimalarial activity with respect to parasitemia progression 17, 29–31

### Histopathological analysis

**Figure 10:**
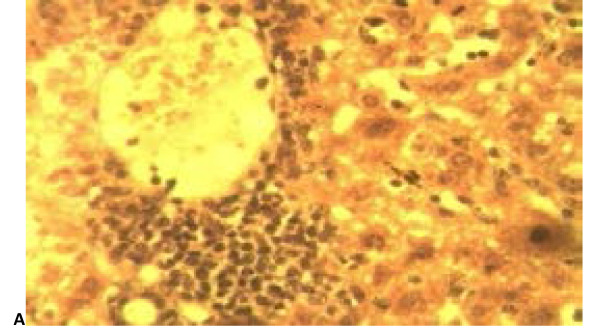
A photomicrograph of liver sections from experimental groups A (M_o_), showing periportal mononuclear cells infiltration. H and E X 400.

**Figure 11:**
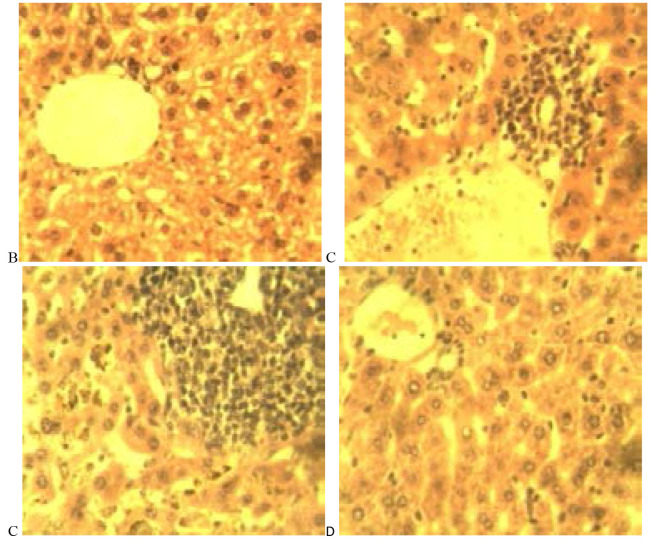
A photomicrograph of liver sections from experimental groups treated with (coartem^®^) (B), (chloroquine phosphate) (C), (with no treatment) (D) and E (no parasite inoculation) showing periportal mononuclear cells infiltration in C and D (white arrows) while there is moderate vacuolar degenerations of hepatocytes (black arrows) and clearance of inflammatory reaction in B. Note the severe hemosiderosis in D (blue arrows). The section in E is apparently normal. H and E x 4.

Table 5 is a summary of the histopathological study carried out on groups treated with the NLC formulation Mo (A), coartem^®^ (B), chloroquine phosphate (C), distilled water (D) and group with no parasite inoculation (E). From the results, it was observed that samples from group D showed severe periportal inflammation while those from groups A and C displayed moderate inflammation while samples from groups B and E showed no sign of periportal inflammation (Figures 10 and 11).

**Table 5:** Summary of histological lesions in the liver of rats from the experimental groups A-E

Lesions/groups	A	B	C	D	E
Periportal inflammation	++	-	++	++++	-
Kupffer cell hyperplasia	-	-	+	++++	-
Hemosiderosis/hemozoin	+	-	+	++++	-
Hepatocytes vacuolation/ necrosis	+	++	-	+	-

Keys: - absent, + mild, ++ moderate, +++ severe, ++++ very severe.

Furthermore, it was also noticed that animals in group D had severe hemosiderosis and severe kupffer cell hyperplasia as well as mild hepatocyte vacuolation. Group A subjects had no kupffer cell hyperplasia but demonstrated mild hemosiderosis and hepatocyte vacuolation. Animals in Group B did not display any periportal inflammation, no hyperplasia nor hemosiderosis was observed but moderate hepatocyte vacuolation was noted. Animals from Group C had mild hyperplasia, mild hemosiderosis and showed no sign of vacuolation. Group E samples showed no sign of inflammation, hyperplasia, hemosiderosis or hepatocyte vacuolation.

From the overall result of histopathology conducted on both liver and kidney of the different groups, it was noticed that, in grading of effectiveness of the different treatments in management of malaria in comparism to the negative control group, coartem® appeared to be the most effective followed by Mo and the chloroquine phosphate.

### Stability of the NLCs

**Figure 14:**
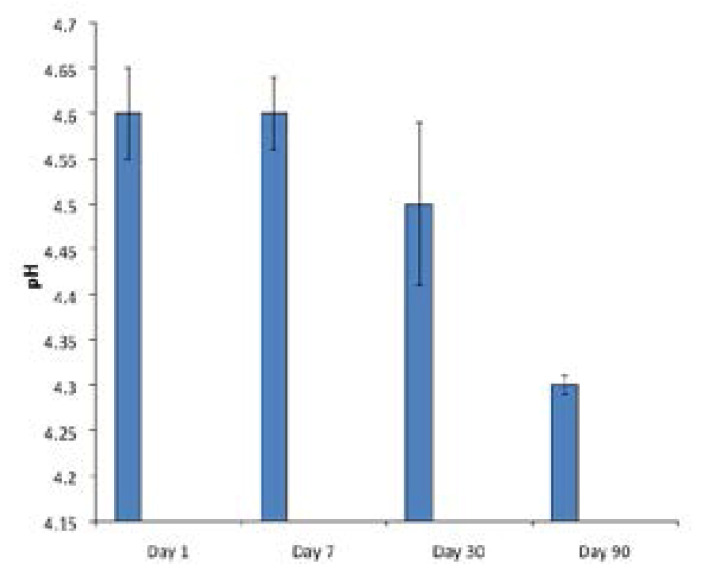
pH variation during storage of the NLC (M_0_).

This test was performed to determine the pH stability of the SLMs batches when stored at room temperature and at different time intervals 23. A good knowledge of the pH of maximum stability of a drug or its stability profile is important, especially in the design and formulation of a stable dosage form of the drug. The information will enable a formulation scientist to decide with certainty whether a stabilizer is necessary or not in the formulation of the drug 23. The pH of the formulation was maintained during the period of storage- one day, one week, one month and three months, as shown in Figure 14, which is an indication of the stability of the formulation, consistent with our earlier report 23.

## Conclusion

In this study, artemether and lumefantrine encapsulated in nanostructured lipid carrier was successfully prepared using caprol-PGE 860.The developed NLCs possessed the desired particle size (mean 188.6 nm) and particle size distribution. The NLC exhibited a better sustained inhibition of plasmodial growth in the malaria infected Wistar mice than the conventional preparation of artemether-lumefantrine in the market sample (coartem^®^ ) and chloroquine. The anti-malarial activities (reduction in parasitemia level, restoration of normal hemoglobin level and restoration of normal packed cell volume) of coartem^®^ and chloroquine phosphate started to reduce after day 7 post treatment but the formulation NLC (Mo) maintained its antimalarial effect throughout the duration of the study. The most important observation is the sustained release ability of the formulation which is an advantage over the conventional tablet (coartem^®^) as it offers us an option of having a formulation with better dosage regimen and more tolerable dose. Efficacy of the prepared NLCs in murine model of malaria infection suggests that this preparation could behave in similar fashion in humans though actual trials need to be carried out to justify this assertion. It may be posited that artemether-lumefantrine NLC is a promising option for use in the global battle to contain the scourge of severe malaria especially in malaria endemic countries.

## Figures and Tables

**Figure 12:**
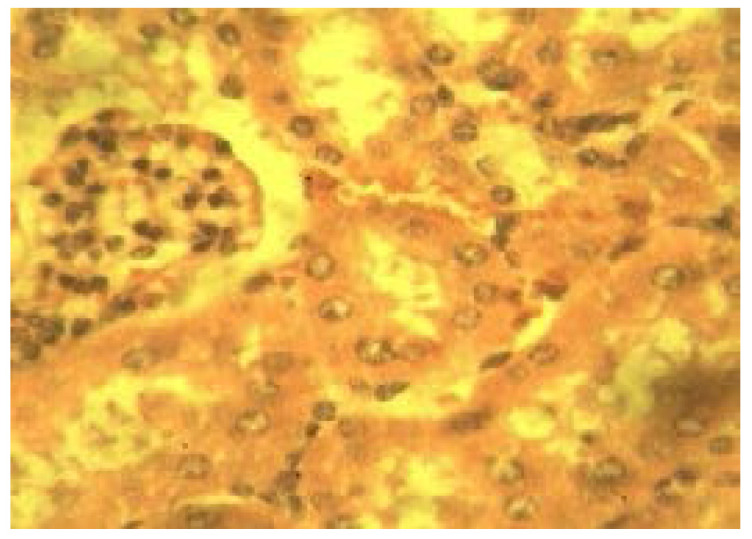
Photomicrograph of sections of the kidney from experimental rats of groups A (M_o_). H and E x 400.

**Figure 13:**
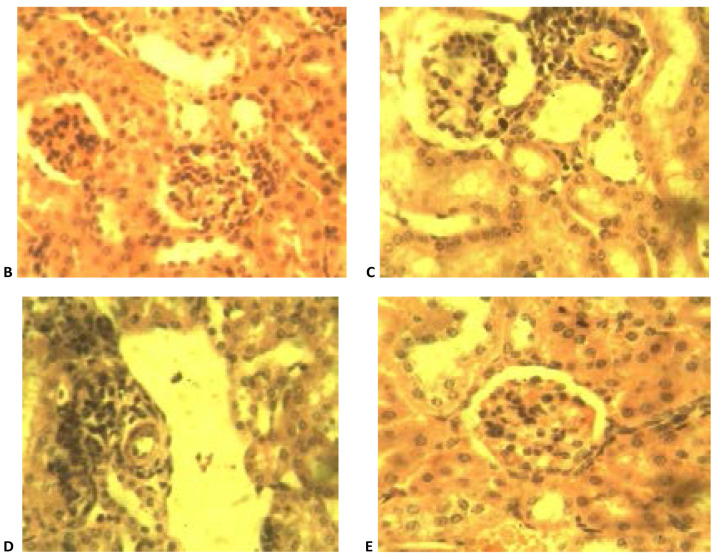
Photomicrograph of sections of the kidney from experimental rats of groups B (coartem^®^), C (chloroquine phosphate), D (distilled water) and E (no parasitemia inoculation) showing perivascular mononuclear infiltration (vasculitis) in C and D (white arrows) while B and E shows no remarkable histologic change. H and E x 400.
